# Contesting the crisis narrative: epidemic accounts in Sierra Leone, Tanzania, and Democratic Republic of the Congo

**DOI:** 10.1111/disa.12535

**Published:** 2022-10-09

**Authors:** Shelley Lees, Luisa Enria, Myfanwy James

**Affiliations:** ^1^ Professor, Department of Global Health and Development London School of Hygiene and Tropical Medicine United Kingdom; ^2^ Assistant Professor, Department of Global Health and Development London School of Hygiene and Tropical Medicine United Kingdom; ^3^ Departmental Lecturer in Development Studies University of Oxford United Kingdom

**Keywords:** Africa, catastrophe, Covid‐19, crisis, Democratic Republic of the Congo, epidemic, ethnography, health, narratives, pandemic, Sierra Leone, Tanzania

## Abstract

Scientists and global commentators watched African countries closely in the early months of the COVID‐19 pandemic, predicting an impending disaster: the virus was projected to overwhelm already weak health systems. These expectations were informed by imaginaries of Africa as an inevitable site of epidemic disaster. This paper draws on accounts from Sierra Leone, Tanzania, and Democratic Republic of the Congo to contrast global catastrophe framings with everyday imaginations and experiences of crisis and crisis management. Utilising ethnographic research, the paper initially explores how COVID‐19 was understood in relation to previous epidemics, from HIV (human immunodeficiency virus) to Ebola, as well as political conflict. It then considers how global crisis narratives both inform and are in tension with everyday collective and personal experiences. The paper brings these empirical reflections into a conversation with theoretical debates on the discursive construction of crisis and its effects, and argues that these tensions matter because crisis framings have consequences.

## نبذة مختصرة

في الأشهر الأولى من جائحة كوفيد‐19 ، راقب العلماء والمعلقون العالميون البلدان الأفريقية عن كثب ، وتوقعوا وقوع كارثة وشيكة حيث كان من المتوقع أن يطغى الفيروس على الأنظمة الصحية الضعيفة من الأساس. هذه التوقعات كانت مستوحاة من تخيلات إن أفريقيا موقع لا مفر منه لكارثة وبائية. تعتمد هذه الورقة على حسابات في تنزانيا,سيراليون وجمهورية الكونغو الديمقراطية لمقارنة هيكلة الكوارث العالمية بالتخيلات والتجارب اليومية للازمات و أدارتها. بالاعتماد على الأبحاث الاثنوغرافية ، نستكشف كيف تم فهم كوفيد‐19 و ما له من علاقة بالأوبئة السابقة ، من فيروس نقص المناعة البشرية إلى الإيبولا ، وكذلك الصراع السياسي. ثم نفكر بعد ذلك في كيفية إعلام روايات الأزمة العالمية بالتجارب اليومية الجماعية والشخصية والتوتر معها. تجلب الورقة هذه التأملات التجريبية في محادثة مع المناقشات النظرية حول البناء الخطابي للأزمة وتأثيراتها ، وتجادل بأن هذه التوترات مهمة لأن إطارات الأزمة لها عواقب.

الكلمات الدالة: أفريقيا؛ نكبة؛ كوفيد ‐19 أزمة؛ جمهورية الكونغو الديمقراطية؛ وبائي؛ الأجناس البشرية؛ صحة؛ روايات. جائحة؛ سيرا ليون؛ تنزانيا

## 摘要

在COVID‐19大流行的最初几个月，科学家和全球评论员密切关注非洲国家，预测一场灾难即将来临，因为他们认为病毒将让本已薄弱的卫生系统全面崩溃。这些预测源自于想象非洲不可避免地成为流行病灾难的重灾区。本文借用坦桑尼亚、塞拉利昂和刚果民主共和国的案例，将全球灾难框架与危机和危机管理的日常想象和危机经验及管理进行对比。我们利用人种学研究，探讨了如何理解COVID‐19与以前的流行病（从艾滋病到埃博拉病毒）以及政治冲突的关系。然后，我们分析了全球危机叙述如何与日常的集体和个人经历产生联系，并与之产生紧张关系。本文将这些经验性反思与围绕危机的话语建构及其影响的理论辩论对话，并认为这些紧张关系很重要，因为危机框架会产生后果。

关键词：非洲；灾难；Covid‐19；危机；民主刚果；流行病；人种学；健康；叙述；大流行；塞拉利昂；坦桑尼亚

## Introduction

In 2015, a panel of scientists and public health experts convened by the World Health Organization (WHO) identified 10 priority emerging pathogens likely to cause severe outbreaks in the near future, including new epidemics of severe acute respiratory syndrome (SARS) caused by a SARS‐associated coronavirus (WHO, 2015). While this panel, to some extent, predicted a SARS epidemic, the narrative of impending catastrophe remained within scientific and public health circles.

Five years later, the COVID‐19 pandemic emerged due to the rapid spread of the SARS‐CoV‐2 virus first identified in China's Wuhan Province, and a public health emergency of international concern (PHEIC) was declared. Following Rosenberg's (1989, p. 2) account of outbreaks' dramaturgic forms—that is, they ‘start at a moment in time, proceed on a stage limited in space and duration, follow a plot line of increasing revelatory tension, move to a crisis of individual and collective character, then drift towards closure’—COVID‐19's trajectory towards crisis was fast (see also Chigudu, 2020a). However, global narratives and understanding of the *kind* of crisis COVID‐19 posed differed and evolved across time and space. As Chigudu (2020a) has noted, viruses like SARS‐CoV‐2 have no meaning in themselves; rather, they become significant in the ways in which epidemics intersect with political, economic, and social aspects of life. Tools such as mathematical models become, in this sense, forms of ‘anticipatory governance’, as they influence policy and shared understandings of future trajectories through their interpretations of past patterns and assumptions about social dynamics (Rhodes et al., 2020).

A report released by the United Nations Economic Commission for Africa in April 2020 predicted that between 300,000 and 3.3 million Africans could die from COVID‐19 (UNECA, 2020). This prediction was based on a number of assumptions: that more than one‐half of the urban population of Africa lives in slums, with poor access to clean water; that the majority of these people are informally employed and therefore cannot work from home; that there are high levels of undernourishment in children, a high prevalence of tuberculosis and human immunodeficiency virus (HIV), and weak healthcare systems; and that countries that have weak economies are unable to continue to meet the costs of lockdown (UNECA, 2020). As a result of such a forecast, early in the COVID‐19 pandemic, a narrative of impending crisis emerged that focused on an inevitable catastrophe looming on the African continent, especially in the global media and in the statements of key actors in global health (Nyabola, 2020). In an interview with CNN (Cable News Network) in April 2020, for example, American philanthropist Melinda Gates expressed her apprehension about the pandemic's disastrous impacts on African countries. Her ‘first thoughts’ were concern about the continent's capacity to respond to the pandemic, and she stated that she foresaw bodies ‘out on the street’ in African countries (Gates, 2020). For Peter Sands of the Global Fund, writing in April 2020, ‘[u]nless extremely fast action is taken, the prospect of an imminent catastrophe in Africa will become real’ (Sands, 2020). Overall, the assumption was that Africa was less well‐equipped than countries in the Global North to respond to the threats presented by the pandemic. These assumptions have been challenged as ‘colonial hangovers’ informed by ideas of Africa as a site of emergency and a destination of emerging infections (Chigudu, 2020a, 2020b; Tilley, 2020). Despite a long history of and high levels of expertise across Africa in managing infectious diseases, it was too often assumed that these countries would have little to offer to the pandemic response (Tilley, 2020).

Such thinking turned long‐standing preconceptions into prophecies of impending and inevitable crisis. Africa, however, confounded this catastrophe narrative in the first year of the pandemic. Initial predictions did not materialise: estimates suggest that around 2.7 million cases and 65,000 deaths occurred in Africa in the first wave of COVID‐19 from March to December 2020, with 85 per cent of these cases concentrated in only nine countries (Salyer et al., 2021). Although the second and third waves have been more severe in some African countries, the pandemic undoubtedly spread significantly slower in Africa than in the rest of the world. Over time, narratives about the course of the pandemic on the continent became more nuanced and models became more cautious, while researchers question factors that may have led to lower infection rates in many countries across Africa, which had been said to ‘puzzle – and worry – experts’ (France 24, 2020; see also Ezeh, Silverman, and Stranges, 2021). Yet, initial responses and expectations matter, notably because they shed light on the assumptions and expectations of (primarily Western) global narratives about African public health, but also because these assumptions have consequences.

We do not suggest in this paper that the pandemic is not of concern to Africa, and we do not purport to explain the continent's trajectory. Rather, we take the global narrative at the beginning of the pandemic of an inevitable African crisis as a starting point and trace how it influenced and interacted with the social meanings and experiences of COVID‐19 in three countries: Sierra Leone; Tanzania; and Democratic Republic of the Congo (DRC). In an ethnographically grounded critique of the way in which an impending crisis was imagined for Africa, we highlight the social and material effects that narratives of crisis have on specific contexts. In this regard, we make three main arguments. First, we challenge this ‘emergency imagery’, situating it in broader critiques that illustrate the limits of seeing ‘crisis’ as a sudden and unpredictable ‘event’ that disrupts normalcy (Calhoun, 2010; Redfield, 2013). Second, we propose that anticipations of an African catastrophe waiting to happen relied on expectations of a continent that is ‘always in crisis’ and that such global imageries do not reflect the reality of everyday and lived experiences in different countries across the continent. Third, we show how the anticipatory nature of crisis imageries about COVID‐19, regardless of their accuracy or otherwise, had significant effects on how the pandemic was managed and experienced. In part, these predictions (and the measures that followed) *created* and *reinforced* complex forms of intersecting social, economic, and political crisis at the local level.

The paper is based on our respective long‐term ethnographic engagement in Sierra Leone (2010–21), Tanzania (2004–21), and eastern DRC (2017–21), where we have conducted research projects to investigate everyday experiences and navigations of crisis, experiences of Ebola and HIV epidemics and associated clinical trials, as well as the impact of COVID‐19 on local health services. We draw on informal conversations, observations, and interviews conducted in 2020–21 with local citizens, healthcare workers, political leaders, and civil society actors in the three countries in order to assess how global narratives of ‘crisis’ both inform and are in tension with everyday collective and personal experiences in these three countries.

These contexts were selected because we were already doing research into everyday experiences of epidemics as the COVID‐19 pandemic began. In Sierra Leone, the paper capitalises on a range of research projects and interactions (at a distance and face to face as dictated by the pandemic), including a project that collected daily diaries from community health workers in Kambia District in May–July 2020, ongoing qualitative research through the Ebola vaccine trials appraising the impact of COVID‐19 on vaccine uptake, and a collaboration with public health officials on the District Health Management Team (DHMT), entailing weekly ethnographic observation reports by a research assistant embedded in the DHMT and the second author's production of weekly social science analysis bulletins for the district's COVID‐19 response, bringing together insights from different projects and providing recommendations. These findings were then supported by field research in January–February 2021 and the analysis presented below was further validated through follow up ethnographic observations in September–December 2021. Comparisons with Ebola stem from two periods of ethnographic fieldwork (14 months between 2015–16 and 5 months in 2017—including participant observation and more than 70 key informant interviews in total) exploring community experiences of the outbreak. In DRC, the paper utilises in particular ethnographic fieldwork conducted in Goma, North Kivu, between October 2020 and March 2021. This involved ethnographic observations in health clinics and around the city, as well as 63 in‐depth interviews and 11 focus‐group discussion with local citizens, health personnel, and political authorities to understand local experiences of the Ebola epidemic, debates surrounding vaccine trials, and experiences of working during the start of the COVID‐19 pandemic. It also takes advantage of the third author's fieldwork on the politics of humanitarian response in eastern DRC, both before and at the start of the Ebola epidemic (2017–18). In Tanzania, the paper draws on a research project that trained and deployed 11 community health workers to amass points of community debate surrounding the pandemic and pandemic response in Dar es Salaam and Bagamoyo and to gather documentary analysis of newspapers and Twitter feeds following political debates on the pandemic response in the country.

Our positionality as Western researchers with long‐term research engagements in these three countries was further complicated by different experiences of the pandemic in Europe. As we spoke to our collaborators and interlocutors in Sierra Leone, Tanzania, and DRC, we did so from a very specific standpoint: having experienced previous outbreaks in these countries and witnessed their response (during Ebola and HIV), we watched COVID‐19 unfold in our home countries with growing concern for our communities, and we adapted our ethnographic approach to an unusual mixture of online communication and travel amid pandemic‐related restrictions. These tensions made the specificity of these countries' experiences of crisis starker and the mismatch between expectations of catastrophe and everyday crisis more visible.

Using this multi‐sited ethnographic fieldwork, we focus on narratives and experiences of the ‘first wave’ of COVID‐19 on the African continent in 2020. As we illustrate, narratives of ‘crisis’ were filtered through local contexts and influenced by specific political histories—‘crisis’ manifested itself and was experienced in different ways. Whereas Sierra Leone and DRC have experienced post‐colonial violent conflict and Ebola epidemics, Tanzania has remained peaceful since independence in 1961, but did experience a prolonged HIV epidemic.

The paper is structured as follows. First, we outline the theoretical literature examining imaginaries of crisis and the actions this labelling facilitates, as well as everyday experiences of crisis, analysing the tensions and interconnections between them. In the case study section, we present three ethnographic vignettes from Sierra Leone, Tanzania, and DRC. Harnessing these ethnographic descriptions, the discussion investigates how global narratives of crisis not only influenced local imaginaries, but also sat in tension with everyday collective and personal experiences of epidemics.

## Crisis imageries and their consequences

To probe how global narratives about COVID‐19 in Africa influenced and contrasted with everyday experiences in these three countries, we take inspiration from longstanding anthropological debates on crisis and its effects. Vigh (2008), for example, questions the utility of understanding crisis as a distinct and isolated period of time, when lives are disrupted and those affected are unable to control the outside forces that affect the choices available to them. In these readings of crisis, emphasis is placed on the temporariness and abnormality of events such as war, disasters triggered by natural hazards, violence, or disease outbreaks (Redfield, 2005; Vigh, 2008). In contrast, Vigh (2008) notes how in many places across the world, people's existence is defined by a constant state of structural violence, including where conflict and poverty dominate everyday life. In these locations, then, we would do well to approach crisis *as context*, a chronic condition, a ‘terrain of action and meaning’ (Vigh, 2008, p. 8), rather than a singular event or moment of rupture. He goes on to argue that this conceptualisation of crisis sheds light on how people navigate their way through volatile environments, where social positions and relations are ‘reconfigured and reshaped in relation to stable instability and chronic crisis’ (Vigh, 2008, p. 13). Here, people continually assess their social environment as well as how they ‘position themselves in relation to others’ in tactical ways (Vigh, 2008, p. 20). Tactical forms of self‐representation and self‐staging thus become a form of social navigation in contexts of crisis: a form of tactical agency in uncertain and disempowering circumstances (Utas, 2005; James, 2022a).

This paper considers the flipside of Vigh's attention to the endemic instead of the episodic. We are interested in exploring how crisis episodes, or rather crisis imaginaries that define specific moments in time *as emergencies*, affect everyday life and the terrains that individuals have to negotiate. Specifically, we are interested in understanding how these imageries (and the realities that they make possible) become embedded in pre‐existing tactics to navigate uncertainty.

Official crisis imageries, in fact, are important not only in themselves, but also because of the social constellations they produce and the interventions they make possible. We ask, for instance, alongside Caduff (2015), what the implications might be of imagining a ‘pandemic, perhaps’, that is, what are the material effects of anticipating catastrophe even if it does not materialise or at least not as expected? In addition, scholars such as Lakoff (2017) and Kelly (2018) have noted the impacts of ‘technical classifications’ that announce that a crisis is taking place and crystallise a dominant interpretation of the *kind* of crisis that is unfolding. Both of their analyses of the Ebola outbreaks in West Africa in 2013–16, chart, from different perspectives, the shifting realms of possible intervention as official crisis imageries changed. A case in point is the declaration of a PHEIC in 2014, which made it possible to fast track clinical research in ways that had previously been impossible. The interventions and measures rendered possible by differing crisis imageries, in turn, can have important consequences for social relations and political identities. During the Ebola response in Sierra Leone, for example, the intersection of community engagement and securitising approaches (such as the militarisation of the response), generated dividing practices between ‘good’ and ‘dangerous citizens’ (Enria, 2019). Crisis narratives and their accompanying ‘interventionary imaginations’ (Enria, 2020) not only reshape the terrains that people have to navigate, but also necessitate that we pay close attention to how people engage with, resist, and appropriate crisis labels and interventions (James, 2022b). Furthermore, moments of rupture and claims to the extraordinary also reveal the more chronic conditions underlying them. Epidemics, for instance, can be seen to act as ‘a breaching experiment, highlighting longer standing fractures [… within] society and giving voice to older narratives of social suffering’ (Enria and Tengbeh, 2022, p. 46; see also Scambler, 2020).

We are interested in showing both how particular narratives about the COVID‐19 pandemic affected the terrains that ordinary citizens had to navigate, and the contestations and reimaginations of the meanings of crisis to which these new constellations gave rise. The aim of this paper is to deconstruct the concept of crisis by paying attention to how people in Sierra Leone, Tanzania, and DRC tried to make sense of global narratives about the pandemic through their everyday experiences and collective memories of other kinds of more or less (extra)ordinary upheaval.

## Local narratives on epidemics

### Sierra Leone

On 25 March 2020, the President of Sierra Leone, Julius Maada Bio, declared a 12‐month‐long state of emergency across the country in view of the anticipated spread of COVID‐19 across the country. At that point in time, Sierra Leone had yet to record a single case of the disease—that would happen five days later. This pre‐emptive state of alertness, prefiguring a near and seemingly inexorable future, situated Sierra Leone in a broader global narrative of collective crisis in the face of a pandemic that was expected to touch every corner of the world. In a sense, however, Sierra Leone was already part of this narrative, assuming a specific role in catastrophic imageries of what impact the pandemic might have on African countries: memories and pictures from the country's Ebola outbreak of 2014–16 supported widespread projections of health systems near collapse and rapid transmission in urban settlements across the continent.

Memories of the West African Ebola outbreak, which resulted in 3,956 deaths in Sierra Leone (CDC, 2016) as well as wide‐ranging and long‐lasting social and economic effects, played a complex role in that country's response to COVID‐19. On the one hand, the pandemic presented a test for the infrastructures of preparedness established in the wake of Ebola. Fast border closures, the rapid activation of district‐and national‐level response teams, assiduous contact tracing, and laboratory capacity were clear legacies of lessons from the past. Indeed, many response measures, such as checkpoints or three‐day lockdowns for house‐to‐house searches, replicated the Ebola model regardless of the different epidemiological characteristics of COVID‐19. Ebola provided a framework with which to understand what was happening in more ways than one. In daily conversations, efforts to remind people of regulations such as social distancing because ‘this is corona time’ were marked by frequent slips of the tongue, as people mistakenly referred to the wrong epidemic: ‘*Dis nar Ebola … no, Corona, tem!*’. Fears and traumatic memories from the previous epidemic undoubtedly compounded global news about the dangers of COVID‐19 in stoking anxieties. Especially in the early months of the pandemic, this led to avoidance of health centres for fear of nosocomial transmission or even of intentional infection by healthcare workers, who once again found themselves at the centre of the long‐standing dynamics of mistrust that had become evident in 2014–16.

In Kambia District, on Sierra Leone's northwest border with Guinea, the announcement of the state of emergency, followed by the first recorded case of an international traveller, was accompanied by a mixture of concern and anticipation (Enria et al., 2020). Public health officials and citizens alike watched the frontier anxiously, expecting the virus as a foreign intruder. Rumours circulated about its true origins, combining with speculation about what might happen if it spread throughout local communities. As the first cases began to be recorded in the district, they were closely scrutinised by community members who were eager and nervous in equal measure to see what this new virus really looked like. ‘Seeing is believing’, as the common refrain goes. If some were already sceptical, the fact that a significant proportion of cases in the town of Kambia were entirely asymptomatic raised suspicions as to what kind of disease COVID‐19 really was. What did it mean to be infected if most were not sick? Were these people really positive? As time went on, then, memories of Ebola took on a different meaning, no longer foreshadowing but serving comparatively to highlight an illness that was more visibly dangerous, more intelligibly a ‘crisis’. Emphasis was placed on a collective memory of ‘seeing’ Ebola patients and dead bodies, whether because they had been family members or because images had been shared on social media, something that did not happen with COVID‐19 victims. Widespread questioning of whether COVID‐19 was ‘real’ settled on the proposition that it may just be a sickness for ‘Whites’, or that at the very least it was not as serious as other diseases.

These narratives contrasting COVID‐19 as ‘fake’ and Ebola as ‘real’ are interesting on different levels. First, they glossed over the fact that the veracity of Ebola itself had been questioned at the time, prompting numerous analyses of the deep structural and historical roots of mistrust during the crisis (see, for example, Wilkinson and Leach, 2015). This undoubtedly changed over the course of the epidemic as the extent of loss and social disruption caused by the Ebola emergency became hard to challenge. Second, the contrast suggests a broader reconsideration of initial fears that COVID‐19 would be as devastating as Ebola, recognition of the different levels of severity of the two diseases. In this sense, the narrative about whether COVID‐19 is ‘real’ may be less about questioning its existence than interrogating the relative risk posed by this new pandemic. Above all, these kinds of discussions and comparisons between two health emergencies point to fluid contestations of what counts (or should count) as a crisis, when, and for whom.

What was, for instance, unquestionably real about COVID‐19 were the restrictions and regulations that accompanied it. Long queues of trucks remained parked for months on end by the closed border, with produce rotting inside and stranded drivers hoping for an imminent reopening. Inter‐district lockdowns similarly threatened livelihoods and food supply chains, while social and spiritual lives were suspended as churches, mosques, and social gatherings were temporarily interrupted. Checkpoints to enforce mask wearing were a particular source of contention, as they were seen to create opportunities to ‘victimise’ those passing by (as a social mobilisation officer put it) and to extort money. Observers pointed to inconsistencies, such as the fact that officers themselves were often unmasked when they imposed fines, or that they would stop people driving alone in their car, to support the notion that public health was not the primary objective of these exercises. In the absence of the projected spike in cases or collapsing health services, contestations of the social and economic impacts of regulations dominated local narratives about what COVID‐19 meant in practice.

The material effects of crisis labelling had already been evident during Ebola, as emergency declarations made possible a militarised response enforcing momentous disruption to sociality. From the perspective of individuals, this was in practice experienced through intersecting layers of crisis and normality. For some, the emergency period was marked by direct encounters with the virus, yet many experienced it through its socioeconomic consequences, all while other more personal or localised types of crisis (such as seasonal flooding or the loss of a child) accompanied more mundane experiences of daily life in the face of upheaval.

COVID‐19 generated an even starker juxtaposition between national claims of emergency, and the restrictions and regulations this made possible on the one hand, and the low visibility of the disease on the other. This contrast needed explanations and these quickly proliferated on social media and in everyday discussions. Again, the narrative repertoire from Ebola was mined with renewed conviction. During Ebola, for example, those who worked for the response were subject to social critique for ‘eating Ebola money’, as this was manifest in the emergence of new homes and conspicuous consumption by those benefiting from an emergency response salary (Shepler, 2017; Enria and Tengbeh, 2022). Four years later, in the face of a pandemic, the link with this past encounter was drawn explicitly in political analyses within Kambia, a district that had experienced Ebola as a government stronghold and now, since a change in government in 2018, was primarily an opposition area. The new government, analyses went, had seen the money that could be made through a crisis, and felt that COVID‐19 was their turn to ‘eat’. They were fabricating numbers, planting positive tests especially for opposition supporters, and ‘coronising’ everything so as to profit from involvement in the global market of the pandemic. The COVID‐19 response was accused of being packed with political allies. Public health officials were aware of these accusations and found them concerning not solely because they highlighted the challenge of engaging communities to build trust in COVID‐19 regulations, but also because the comparison between a well‐funded and internationally supported Ebola response and the realities of an under resourced and mostly local COVID‐19 response generated expectations that could not be fulfilled, and sparked tensions within the response and between response workers and their communities.

If COVID‐19 had initially been met with anticipation, both through fears of the suffering it might cause and hopes that new opportunities may emerge, a year on as the state of emergency was lifted in March 2021, neither imagery was fully realised for Kambians. Although, to date, severe illness and deaths were much less numerically significant than in the West, the pandemic nonetheless materialised other kinds of crises, upending livelihoods and giving new life and language to longstanding contestations of political legitimacy. Both parallels and disjunctures with the experience of the Ebola epidemic allowed Kambians to articulate this dissonance.

Global narratives about COVID‐19, filtered through the lens of local histories and experience, had significant effects on how Kambians engaged with and experienced the pandemic, first stoking fears and anticipation, and then stimulating debates about what this ‘crisis’ really meant. The Sierra Leonean government's participation in this global narrative of emergency undoubtedly made it possible to put in place rapid response measures that would not have otherwise been possible, but in so doing, also generated contestations about the social effects of these measures, their political significance, and their contrasts with other forms of more endemic causes of uncertainty.

### Tanzania

On 16 March 2020, the Ministry of Health of Tanzania announced the first case of COVID‐19, but a state of emergency was never declared. Instead, by 23 March, President John Magufuli was criticised internationally for encouraging people to continue attending places of worship. While he urged Tanzanians to take precautions, he underscored that: ‘We are not closing places of worship. That's where there is true healing. Corona is the devil and it cannot survive in the body of Jesus’ (Edwards, 2021; see also Tarimo and Wu, 2020).

Magufuli did call for a ban on all public gatherings and closed schools, and suspended football matches and all international flights (The Citizen, 2020). However, public transportation continued to run, and despite the government's call to avoid unnecessary handshaking, concerns emerged that these limited measures would not contain the epidemic and the virus would spread rapidly in the densely populated cities (Edwards, 2021).

Just 509 cases and 21 deaths were reported by May 2020, and the country has not reported any cases of coronavirus since. By April 2020, all public health measures were stopped and schools were opened. Health Minister Dorothy Gwajima announced in February 2021 that Tanzania would not accept COVID‐19 vaccines. This followed an announcement by the president that he did not trust vaccines sourced abroad, without certification by Tanzanian experts. At this televised meeting, unmasked officials instead drank COVID‐Organics—a tonic drink made from a medicinal plant used in traditional medicines—promoting natural methods to kill the virus. Magufuli, in a number of speeches, extended his distrust to other vaccines, stating in 2021 that: ‘You should stand firm. Vaccinations are dangerous. If the White man was able to come up with vaccinations, he should have found a vaccine for AIDS by now’ (Makoni, 2021). Opposition leaders challenged both the denial of COVID‐19 and the rejection of vaccines, depicting the latter as a way to justify the denial of COVID‐19 during the second wave, when deaths were becoming more prominent (Makoni, 2021).

Unlike the other case studies, Tanzania has not experienced an Ebola epidemic, despite bordering DRC and Uganda. However, under the Magufuli government, Tanzania was criticised by WHO for withholding information on suspected cases of Ebola in 2019. When the government insisted that a Tanzanian doctor, who died after returning from Uganda had tested negative, WHO issued an extraordinary statement in September 2019, urging the country to provide samples from suspected cases for testing (WHO, 2019). It is unclear to what extent this management of a suspected Ebola outbreak affected citizen trust in the government. Previous to this, Tanzania had been devastated by HIV since the mid‐1980s. The emergence of this epidemic coincided with the World Bank imposing a structural adjustment programme, which demanded neoliberal economic reforms and the contraction of state health and welfare services. At this time, neoliberal policies allowed for the growth of the nongovernmental sector, which expanded rapidly to provide HIV treatment and prevention services (Dilger, 2003; Beckmann and Bujra, 2010). Essentially, the COVID‐19 pandemic was the first time that the government had intervened directly with public health responses.

#### Public discourse/private narratives

Initially, Tanzanians conformed to the mild public health measures. However, as few people experienced the disease or knew of others who got sick, very rapidly a narrative emerged that COVID‐19 was a ‘White man's’ (*ugongwa wa wazungu*) disease. As the number of cases rose in Europe and the United States, this narrative was verified. Magufuli's rejection of foreign expertise was accepted alongside an ongoing distrust of foreign involvement in medical research. Previous anthropological research as part of HIV trials exposed narratives circulating within communities surrounding the research sites, that foreigners had nefarious intentions to harm Tanzanians (Lees and Enria, 2020). Notions that the HIV virus or ‘White man's’ sperm was injected into HIV prevention technologies dominated. Similar ideas emerged about potential COVID‐19 vaccines, especially the globally circulating rumour that Bill Gates, the co‐Founder of Microsoft, had inserted a microchip into the vaccine (Goodman and Carmichael, 2020). Magufuli further entrenched distrust in foreign science by announcing that he had sent samples from a papaya, a goat, and a sheep to be tested and suggested that the positive results showed that the tests were not valid (AFP, 2020).

Community discussions and online debates moved quickly to accept, with relief, that the impending catastrophe predicted by Europe had not come to fruition. Instead, they focused their concern on the situation in Europe, extending sympathy for European lives in lockdown. At this point, with relief, many suggested that COVID‐19 must be a rich person's disease and therefore Tanzania had escaped this burden. Tanzanians returned their children to school and returned to their workplaces. Yet, despite denial by the president, COVID‐19 concerns remained. Where possible, Tanzanians wore masks and washed their hands. In many ways, this looming virus was experienced like that of HIV: a potential threat to life, but not a catastrophe.

As the elections in November 2020 approached, public experiences of the pandemic were muted by governmental control of the media and social media. Nevertheless, stories emerged of night‐time burials and opposition leaders, as well as scientists and public health officials, endeavoured for transparency and reporting of cases and deaths. By the end of 2020 and into early 2021, a number of public deaths from pneumonia‐type illnesses become public. The death from COVID‐19 of the Vice‐President of Zanzibar, Seif Sharif Hamad, and other high‐ranking government officials could not be denied in public and public narratives now focused on the acceptance that there was a burgeoning epidemic. When Magufuli suddenly stopped appearing in public from 27 February 2021, rumours quickly emerged that he was ill with COVID‐19. Secrecy surrounded his disappearance until 17 March when his death from a heart condition was announced. Since his passing, debates about the extent of COVID‐19 and challenges to his denialism have continued on social media. The new president, Samia Suluhu Hassan, set up an expert taskforce on COVID‐19 to advise her government and vaccines are being rolled out. Reporting of cases has recommenced with numbers reaching 33,000 and deaths of around 800 as of January 2020 (Our World in Data, 2022).

### Democratic Republic of the Congo

On 10 March 2020, DRC registered its first COVID‐19 case. By the end of the month, there were 40 cases in the capital, Kinshasa. President Félix Tshisekedi announced a national lockdown: schools, bars, restaurants and places of worship were closed, travel to and from Kinshasa was banned, and international flights were suspended. North Kivu, a province situated 2,400 kilometres east of Kinshasa, was in the midst of the country's tenth Ebola outbreak. Now, it seemed, a new ‘crisis’ was on its way.

#### From anticipation and confusion to contestation

As COVID‐19 cases soared in Europe, WhatsApp videos of overwhelmed French hospitals uploaded by global news outlets went viral in Goma. People described a sense of impending doom: ‘we watched on France 24 the crisis unfold, and we thought, if that's what is happening in Europe, where health systems work, what is COVID‐19 going to do to us here?’.^1^ The swift imposition of a lockdown by the government reinforced the impression that an unprecedented catastrophe was on its way, and there was panic as a local hospital released a statement confirming that it had no ventilators. One doctor told us that in the end, he bought as much canned food as he could, assembled his family, and waited.

But the hospitals did not immediately fill up. The predicted catastrophe did not unfold as anticipated. People already knew what the COVID‐19 ‘looked like’: they had seen it on France 24. So, where was it? Some concluded that COVID‐19 exists in Europe, but not in Africa, highlighting differences in demographics, climate, food, travel, and traditional medicines. For others, it was a case of ‘strong blood’. One humanitarian explained: ‘It is because Africans are used to suffering since we were children. Whites are spoilt! This is your first experience of suffering for years, so you are weak’.^2^ A doctor concluded, ‘your immune system was sleeping, but ours was always awake’.^3^

Others determined that COVID‐19 was not a global health crisis, but a moral crisis that targeted the rich and powerful. God had gifted Africans with immunity and was punishing Westerners for colonialism. Meanwhile, in DRC, COVID‐19 was a Robin Hood virus, targeting the rich and mobile: ‘God is the protector of the weak’, one colleague told us, ‘Kinois were saying look at me I just got back from Europe, and then they got COVID. Then the elite here were saying look I'm visiting family in Kinshasa, and then they got COVID too’.^4^ One student argued that even in the capital, COVID‐19 does not exist. To explain why people had died, he said that travel restrictions meant that ‘big men’ could no longer fly to Europe for specialised healthcare. They died because they had to use the dilapidated local health system like everyone else.

Many grappled with the lack of visibility of the crisis: they had not *seen* COVID‐19, or anyone who had died of it. Even those who tested positive showed no symptoms. COVID‐19 was compared to the free Facebook application: you see what is written, but you cannot see the images. You hear about it, but you do not see it. This analogy suggested that things were being hidden. For many, the explanation was that just like Ebola, COVID‐19 was not a health crisis but a ‘cop’: a business. One friend summarised: ‘The government inflates numbers to attract donor funding. Doctors are paid to say someone died of COVID‐19 so that the business can grow’.^5^ COVID‐19 was either invented, or exaggerated, to replace ‘Ebola business’.

The Ebola epidemic ended in June 2020: approximately USD 1.2 billion was spent during the outbreak (Crawford and Holloway, 2021, p. 41). The introduction of this response in an area where basic services are underfunded and where there has been a failure to prevent recent massacres of civilians gave the impression that it aimed to enrich local authorities and responders, rather than help local communities. The response largely sidelined local health systems and staff, and corruption and inflated salaries for outsiders made people believe that the responders had incentives to prolong the outbreak, or even invent Ebola altogether. In 2018, elections were cancelled in the region: Ebola was also seen as a scheme to suppress the opposition stronghold.

COVID‐19, then, was a way to continue the business. However, this time, ‘the partners’ (non‐governmental organisations) in the West were suffering. Therefore, the ‘numbers magically got better when the government realised they couldn't attract funding’, one businessman concluded.^6^

COVID‐19 was also described as a business for Westerners: there were hidden interests at play. Rumours circulated that Bill Gates had *created* COVID‐19 in order to then make money from providing a vaccine. COVID‐19 was thus another way for Western institutions to continue making money by supplying ‘cures’ to Africa, while sidelining local medicines that threatened ‘Big Pharma’ interests. There was a public outcry when a Congolese scientist stated that DRC was a ‘candidate’ for future COVID‐19 vaccine trials: he had to reassure the country that they would not be used as ‘guinea pigs.’ Meanwhile, people in Goma spoke highly of the President of Madagascar, Andry Nirina Rajoelina, for promoting an ‘African solution’: a tonic drink called COVID‐Organics made from artemisia, a medicinal plant used in traditional medicines for millennia. There was much pride in Jérome Munyangi, a Congolese doctor who was involved in COVID‐Organics. A young man working in rural development explained: ‘people find COVID‐19 Organics hard to believe. Medicines always have to be from WHO, or White people. But we also have well‐qualified people. We also have capacities, but they are trampled on’.^7^ A traditional healer concluded an interview by stating that colonisation is ‘the monopoly on invention and innovation’ and that there is nothing more colonised than medical research. He asked whether we might return to Europe and tell people that Africa does not just have problems, but also solutions: ‘things to offer the world’.^8^

#### ‘The crisis is the quarantine’

For many, the ‘crisis’ was not the virus itself, but the *impact* of public health measures themselves. ‘The crisis is the quarantine. It is not a health crisis, it is a political crisis!’, one teacher summarised.^9^ COVID‐19 was experienced as another layer of structural violence in a continued crisis of governance. In November 2020, Tshisekedi dissolved the government and started ‘consultations’ in an attempt to reform a parliamentary majority. In 2019, he had taken over from Joseph Kabila after long‐delayed elections. But Tshisekedi's rise to power depended on a coalition with Kabila's allies. In 2020, Tshisekedi attempted to break from his predecessor. During this political turbulence, lockdowns and a curfew were interpreted as tools to prevent protests. Tensions grew in early 2021 as schools and universities remained shut, while bars and restaurants (popular among the middle class and foreign aid workers) were reopened. People explained this inconsistency by the fact that Tshisekedi had promised free primary school, ‘but he's eaten all the money!’. Others criticised the police's enforcement of health restrictions. One young man summarised that COVID‐19 ‘has become a cop to arrest us, instead of asking us for our identity cards, police now just ask us: “Where is your mask?”’. Another underlined: ‘We are not afraid of COVID, we are afraid of the police. Our state governs badly. They closed the schools, and then police harass people and demand money if they don't have a mask.’^10^

COVID‐19 was also described as a continued form of structural violence from outside the continent—an extension of Western colonialism. After the deaths of two COVID‐19‐sceptic African presidents (Pierre Nkurunziza in Burundi and John Magufuli in Tanzania), rumours circulated that they had been killed for challenging the West's COVID‐19 business. As one colleague asked: ‘Why is it that it's only African leaders who oppose COVID who die?‘. A *tshukudu* driver intimated that the West continues to remove anyone who goes against it, such as Patrice Émery Lumumba, DRC's first president, who was assassinated on 17 January 1961.^11^

COVID‐19 was instead experienced daily as a set of personal crises provoked by the lockdown restrictions themselves. The ‘copy and paste’ approach from Europe was widely criticised: ‘People can't stay at home, they need to go out in order to eat!’, one civil society activist said;^12^ a student added, ‘COVID created a psychosis in the population because it made people so poor by the fact that they could no longer do their daily activities’.^13^ Goma is situated on the border with Rwanda, which is crossed by thousands of people every day. COVID‐19 restrictions shut the frontier and trade stopped. One trader explained: ‘COVID affected us because we depend on daily business, I buy tomatoes in Rwanda and sell them here. But when they shut the borders, it was impossible’.^14^ To make matters worse, with crossing points shut, local food prices soared. Others highlighted the psychological impact, depicting COVID‐19 as ‘traumatising’: ‘I have never seen schools and churches shut for so long’, one woman stated.^15^

Yet, once the lockdown was lifted, people described a degree of continuity: COVID‐19 became another element that added to, and exacerbated, the existing context of prolonged ‘crisis’. ‘After Ebola, COVID arrived, nothing really changed’, one healthcare worker noted.^16^ In the city, public health infrastructures were converted: COVID‐19 stickers were stuck next to Ebola stickers on water tanks and check points about taking one's temperature, while COVID‐19 posters joined fraying Ebola posters in health clinics. During conversations, people talked about COVID‐19 and Ebola interchangeably and sometimes misspoke. They pointed to the degree of continuity between the two: ‘COVID is similar to Ebola for us. You need to wash your hands, for Ebola we were also asked to wash our hands, you need to wear a mask, also for Ebola they asked us to do that. People think it's the same thing’, one young man remarked.^17^

Ultimately, COVID‐19 and Ebola never ‘arrived’ in the way that was anticipated in Goma: the city had only a few cases of Ebola. Instead, Gomatraciens lived in anticipation: ‘now they say its COVID which is coming, but I've still never seen anyone die of COVID. In the end, I never saw anyone die of Ebola either’, one teacher told us.^18^

## Rethinking ‘crisis’

In these vignettes, we deconstruct what ‘crisis’ means in everyday life and illustrate how it is always layered, entangled with social and political histories, with complicated interconnections between public and private experiences. We focus on the start of the COVID‐19 pandemic to highlight the social life of narratives of a crisis that had not yet arrived. In all three cases, the narrative of inevitable crisis did not come to fruition in the ways expected. Global narratives of crisis both influenced local imaginaries of the event, but also were contested by those who could not see COVID‐19's immediate and catastrophic impact on their daily life. International news stories and videos shared on social media created a sense of anticipation. Yet, when everyday experiences did not match this crisis imaginary in Sierra Leone, Tanzania, and DRC, many began to doubt COVID‐19's presence at all. In a broader context of mistrust and scepticism, visibility—*seeing* crisis with one's own eyes—was crucial.

The way people engaged with and experienced COVID‐19 was shaped by recent experiences of Ebola in Sierra Leone and DRC, but in slightly different ways. In Kambia District in Sierra Leone, traumatic memories of *seeing* Ebola victims heightened local anxiety and increased mistrust in the face of COVID‐19's invisibility. In Goma, DRC, however, many inhabitants had not seen Ebola victims themselves—the epidemic had unfolded further north in the province, with only a few cases in the city itself. The invisibility of COVID‐19, then, was seen as an exaggerated version of these dynamics of invisibility—a virus that remained invisible even among those who tested positive. The conclusion that COVID‐19 was just another ‘business’ to serve the economic and political interests of the powerful communicated deeper anxieties and historical experiences in Sierra Leone and DRC (White, 2000). It drew on imperial experiences and medical histories: biomedicine was a central instrument of control, with Africa seen as a ‘living laboratory’ (Tilley, 2020). This narrative of COVID‐19 business also drew on recent experiences of global health governance during Ebola epidemics and several Ebola trials to test vaccines designed by Western pharmaceutical companies. Critiques of emerging ‘businesses’ became an astute political commentary of the recent role of epidemic response in the local political economy, the forms of exclusion and inequality it reproduced, as well as the continued neglect of priorities such as insecurity, a lack of basic services, and treatment for other (more deadly) diseases that did not pose a potential security threat to ‘the West’ (James, Kasereka, and Lees, 2021). The focus on a largely invisible threat, while real priorities continued to be overlooked, led to the conclusion that COVID‐19 was another instrument for further extending state and foreign power. Indeed, anticipatory regimes encouraged disease exceptionalism, the maldistribution of treatments, and reproduced hierarchies of slow‐building institutions (Benton, 2015; Groupe d'étude sur le Congo, 2020).

Contesting global narratives of crisis also became a means of challenging the depiction of Africa as a site of inevitable catastrophe and articulating a pan‐African form of resistance to Western medicine's global hegemony (Richey et al., 2021). This time, people stressed, it was ‘the White man's’ turn to experience a crisis. Just as ‘traditional’ practices held social and political value as a form of anticolonial resistance in the colonial era (Hunt, 2016), championing traditional cures to COVID‐19 (and sometimes challenging the introduction of vaccines) was also a means of critiquing global philanthro‐capitalism, which continues to exclude certain knowledge and actors, defining some people as traditional and particular, in contrast to universal and scientific. It became a way to champion African success stories, which are erased within global institutions (Tilley, 2020).

COVID‐19 presented simultaneous continuity and rupture. In DRC, for instance, the narrative that COVID‐19 had not changed that much acted as a political critique: things have not improved. The fact that in DRC and Sierra Leone people often confused Ebola and COVID‐19 illustrates the sense of continuity between the two experiences. Rather than an exceptional event, COVID‐19 became entangled in an existing context of political turbulence and uncertainty, as well as cyclical epidemics. Eastern DRC, for example, has experienced prolonged conflict for more than 20 years. After decades of economic downturn, political volatility, and protracted violence, people have developed improvisational methods for manoeuvring through an informal economy and complex social terrain in DRC (Trefon, 2004). People live by the ethos of *débrouillardisme*, which means ‘muddling through’. COVID‐19 became the latest addition to an existing context—another set of structures to navigate (Vigh, 2008). As with Ebola, it revealed and exacerbated troubled relations with state and society, as citizens rearticulated their dissatisfaction with the prolonged presence of aid actors, which many saw as complicit in broader processes of exclusion and oppression. Collectively, it was similarly experienced as a manifestation of broader political crises of governance and inequality.

Yet, COVID‐19 also represented a change to the existing context. Although it did not materialise as the narrative of catastrophe suggested, global crisis narratives did have important social and economic ramifications for peoples' lives. In effect, narratives of crisis and the labelling of ‘crisis’ did important work because they made new types of action and intervention possible (Enria, 2019). Political authorities in Africa implemented pre‐emptive public health measures for a global crisis that did not yet necessarily directly and visibly affect the lives of their citizens. Central states enforced unprecedented lockdown measures after only a few cases had been registered. Some of these may have helped to curb infections, but border closures, the shutting of schools and churches, quarantine, and the criminalisation of people who did not follow these measures also altered lives and created more nuanced forms of everyday crisis that intersected in complicated ways with existing forms of structural violence. People did not experience the health crisis depicted in global narratives during the first wave of the pandemic, but complicated social, economic, and political crises caused by the restrictions themselves. Rather than conforming to the ‘emergency imaginary’ (Calhoun, 2010) of an unpredictable and sudden event disturbing normalcy, the impact of COVID‐19 was experienced as slow, structural, and deeply political.

‘Crisis’ was filtered through local contexts: it manifested itself and was experienced in different ways in these three settings, influenced by political histories and a series of other, complex overlapping ‘crises’. The imaginaries of inevitable crises were contested in public and political debates in Tanzania, Sierra Leone, and DRC, but in different ways. In Sierra Leone and DRC, narratives surrounding COVID‐19 were shaped by recent experiences of Ebola epidemics and to some extent, a preparedness for future epidemics. In contrast, Tanzanian debates were influenced strongly by the government's narratives that rejected foreign involvement. While the HIV epidemic in Tanzania was principally addressed by international and national non‐governmental actors at its emergence, high‐level government involvement was immediate in the suspected Ebola outbreak and the rapidly emergent COVID‐19 pandemic. In local narratives in Tanzania, many citizens initially supported the president's stance against foreign involvement, possibly because of a longer history of HIV medical research and response (Lees and Enria, 2020). This contrasted with a more critical reaction to the DRC's government, which was accused of ‘copying and pasting’ foreign public health measure for political gain. In DRC and Sierra Leone, contesting the ‘COVID‐19 crisis’ was an everyday narrative among citizens, a political and social critique of existing structures of governance, as people accused the governments of the two countries of creating COVID‐19 cases for business. Meanwhile, the contestation of crisis in Tanzania was situated in the government itself, with its opposition and some vocal citizens accusing it of *underplaying* the number of cases and deaths.

## Conclusion

The narratives of crisis and their global imaginaries both shape local imaginaries of crisis and are in tension with local experiences. The COVID‐19 pandemic presented simultaneous forms of continuity and rupture. In Sierra Leone, Tanzania, and DRC, the global imaginary of crisis and catastrophe in Africa was not realised during the first wave of the pandemic. However, the anticipatory nature of crisis imageries about COVID‐19 had significant effects on how the pandemic was managed and experienced, especially at the political level. In part, these predictions and the subsequent measures *created* and *reinforced* complex forms of intersecting social, economic, and political crises at the local level. In addition, the labelling of crisis and this anticipatory regime encouraged disease exceptionalism, and reproduced forms of exclusion by overlooking other more pressing local priorities, not only in terms of health, but also insecurity, a lack of basic services, political repression, and state neglect.

Ultimately, a global narrative of crisis facilitates such vertical, top‐down approaches. As MacGregor et al. (2021, p. 4) argue, ‘response plans for epidemics that privilege top‐down action have been the norm, and governments have frequently echoed restrictive measures for COVID‐19 as implemented in other global regions’. In preparing for future pandemics, there is a need for deeper understanding of the complex lived experiences of ‘crisis’, which are shaped by, but also are in tension with, these narratives of global catastrophe. Closer attention to complex, layered, and deeply contextual experiences is crucial to ensuring epidemic responses that take into account the social, economic, and political specificities of different contexts.

## Acknowledgements

Part of this research is funded by the Department of Health and Social Care using UK Aid Direct funding and is managed by the Biotechnology and Biological Sciences Research Council, the Engineering and Physical Sciences Research Council, and the National Institute for Health and Care Research. The views expressed in this paper are those of the authors and do not necessarily represent those of the Department of Health and Social Care. Shelley Lees and Luisa Enria wish to acknowledge funding from EBOVAC3 funded by the Innovative Medicines Initiative 2 Joint Undertaking under grant agreement number 800176. This Joint Undertaking receives support from the European Union's Horizon 2020 research and innovation programme and the European Federation of Pharmaceutical Industries and Associations. Luisa Enria also wishes to acknowledge funding from the Economic and Social Research Council (Fellowship No. ES/N01717X/1). Myfanwy James would like to point out that this paper draws on research supported by the Coalition for Epidemic Preparedness Innovations, Ebola Vaccine Deployment, Acceptance and Compliance, and UK Research and Innovation. We wish to thank our colleagues and interlocutors across the three countries.

## Data availability statement

The data that support the findings of this study are available from the corresponding author upon reasonable request.

## Endnotes

1 Comment to the authors, Goma, October 2020.

2 Comment to the authors, Goma, November 2020.

3 Comment to the authors, Goma, February 2021.

4 Comment to the authors, Goma, January 2021.

5 Comment to the authors, Goma, November 2020.

6 Comment made in a focus group, Goma, November 2020.

7 Comment made in a focus group, Goma, November 2020.

8 Comments made in an interview, Kilijiwe, November 2020.

9 Comment made in an interview, Goma, February 2021.

10 Comments made in interviews, Majengo, October 2020.

11 Comments made in a focus group, Goma, March 2021. For more information on the Lumumba example, see Soudan (2021).

12 Comment made in an interview, Goma, October 2020.

13 Comment made in an interview, Majengo, October 2020.

14 Comment made in an interview, Kahembe, October 2020.

15 Comment made in an interview, Majengo, October 2020.

16 Comment made in an interview, Goma, February 2021.

17 Comment made in an interview, Majengo, November 2020.

18 Comment made in an interview, Majengo, October 2020.
